# Exploring clinical teachers’ beliefs about teaching in a newly established medical school in Southern Switzerland

**DOI:** 10.1186/s12909-024-05299-0

**Published:** 2024-03-22

**Authors:** Marilù Guigli Poretti, Matteo Monti, Marta Fadda

**Affiliations:** 1 Ente Ospedaliero Cantonale, Area Formazione Medica e Ricerca, Direzione Generale, Lugano, Switzerland; 2 Medical Education Unit, Faculty of Biomedical Sciences, Università della Svizzera italiana, Lugano, Switzerland; 3 Medical Education Unit, Faculty of Biology and Medicine, University of Lausanne, Lausanne, Switzerland; 4 Division of Internal Medicine, Lausanne University Hospital, Lausanne, Switzerland; 5 Institute of Public Health, Faculty of Biomedical Sciences, Università della Svizzera italiana, Lugano, Switzerland

**Keywords:** Medical education, Normative beliefs, Behavioural beliefs, Self-efficacy, Faculty development

## Abstract

Academic social contexts and educational beliefs may influence teaching practices and teaching intentions. Insight into such beliefs represents an important source of information for medical schools to improve the quality of teaching and to guide content of faculty development programs. The aim of this study was to explore beliefs about teaching among prospective clinical teachers at a newly established medical school in Southern Switzerland and to estimate the relationship between these beliefs and intention to commit to teaching in the newly established medical curriculum using Fishbein’s integrative model of behaviour prediction. We targeted a purposive sample of physicians working in hospital departments potentially involved in the clinical immersion of medical students enrolled in the program. We designed a cross-sectional quantitative study using an online questionnaire. The questionnaire included both items developed by the authors and items belonging to a previously validated questionnaire. Participants showed a high intention to commit to teaching in the newly established curriculum. We found that self-efficacy beliefs and two sets of behavioural beliefs, namely perceived importance and expectations, had a positive correlation with the intention to commit. On the other hand, we pointed out a number of normative beliefs that in the long run could hinder the maintenance of this commitment, which should be addressed both at the levels of both faculty development and institutional policy rules. Our study also highlights the importance of reinforcing teachers’ perceived self-efficacy in providing clinical supervision, strengthening their perceived importance of the clinical supervision, and incentivising the commitment of teachers by ensuring they have protected time to devote to this activity.

**Supplementary Information:**

The online version contains supplementary material available at 10.1186/s12909-024-05299-0.

## Introduction

Providing high quality education to future physicians is vital for maintaining high standards of healthcare systems [1, 2]. Clinical teachers play a central role in this effort. The current competence-based medical education framework requires high standards for teachers in teaching, learning facilitation, role modelling, assessment of progression and feedback skills [2–4]. Clinical teaching aims to integrate medical knowledge into professional activities, develop clinical reasoning skills, and motivate students. Clinical teachers are expected to reflect on their teaching, and act on feedback [5, 6]. However, factors such lack of time, motivation, and skills, and personal beliefs about teaching may influence clinical teachers’ actions and intentions [7, 8], making the provision of clinical supervision suboptimal and extremely variable [9, 10]. Understanding clinical teachers’ beliefs can guide faculty development and improve the quality of education [7].

Various studies examining the educational beliefs of educators have introduced classification rubrics that categorize beliefs along a continuum, delineating global orientations from teaching-centred to learning-centred perspectives [11–18]. Teaching-centred beliefs emphasize the transmission of specific content or knowledge, while learning-centred beliefs prioritize students’ conceptual understanding and development. While these classification frameworks provide a structured approach to differentiate between educational beliefs, offering valuable insights into crucial aspects of pedagogical ideologies, their predominant emphasis on the teaching-learning continuum tends to overlook other relevant dimensions of beliefs regarding education, such as normative and behavioural beliefs. Moreover, the application of these classification frameworks in the field of medical education remains limited. Finally, there exists a notable gap in comprehending the intricate interplay between medical teachers’ beliefs and critical drivers of action, such as intention and its determinants, including self-efficacy, within the specific context of clinical teaching. While individual studies have independently explored these elements, a comprehensive synthesis of their collective impact on clinical teaching is lacking. This study seeks to address this gap by conducting a nuanced exploration of medical teachers’ beliefs and their intention to commit to teaching within the realm of clinical teaching, thereby contributing valuable insights to the existing body of knowledge.

In addition, while there is a growing body of literature on medical education in various global contexts, there is a limited exploration of the specific challenges and dynamics encountered by clinical teachers in the Swiss context. By providing a detailed account of the situation within one of the medical schools in Switzerland, this study seeks to enhance the generalizability of findings and offer a context-specific perspective on medical teachers’ beliefs and their role on clinical education. Recognizing the influence of cultural and institutional factors on clinical teaching, the study aims to fill a crucial gap in the literature by offering insights that are directly applicable to the Swiss medical education system, while also contributing to the broader discourse on clinical teaching worldwide. Undergraduate medical education in Switzerland lasts for 6 years: 3 years of Bachelor, with a focus on basic science, and 3 years of Master, more devoted to clinical studies. In 2019, the Università della Svizzera italiana (USI) in Southern Switzerland established its own medical school, opening its doors to the first cohort of Master students in September 2020. Most of the students had completed their Bachelor in another Swiss medical school. The USI curriculum includes a substantial amount of clinical immersion. For most physicians hired into the program this represented their first experience to provide clinical training to medical students and a different curricular model compared to the traditional one they encountered during their medical schooling. The aim of this study was to predict behavioural intentions of future clinical teachers at USI, by exploring their beliefs about teaching and their intention to commit to teaching in the newly established medical curriculum. Results will be helpful to build a faculty development program targeting specific teachers’ needs.

### Theoretical framework

**Fig. 1:**
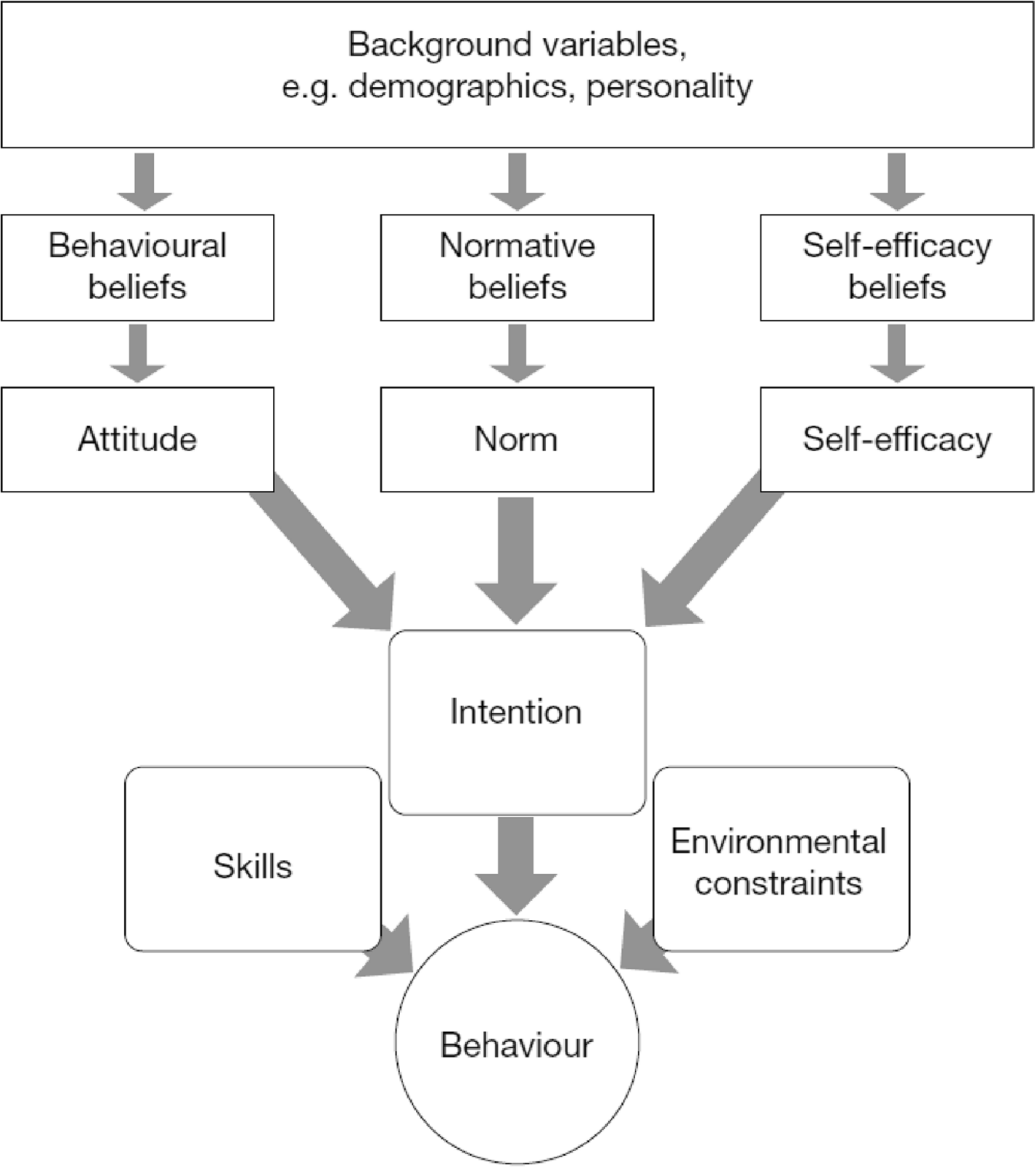
Fishbein’s integrative model of behaviour prediction (IMBP). Reproduced with permission from Dory et al. (2015)

Fishbein’s integrative model of behaviour prediction (IMBP) provided the theoretical framework for this study (see Fig. 1) [19]. The IMBP posits that behaviours are driven by the intention to perform the behaviour, the ability to perform the behaviour, and environmental constraints. Intention is in turn determined by behavioural, normative, and self-efficacy beliefs. **Behavioural beliefs** are expectations about the potential costs and benefits of the behaviour. In this study, we conceptualized behavioural beliefs in terms of (a) clinical teachers’ *expectations related to teaching* in the medical curriculum, (b) the extent to which participants *identified themselves in the role of a clinical teacher*, and (c) the *perceived importance* of clinical supervision. We further distinguished between expectations in relation to clinical teachers’ work performance, their relationship with colleagues, and their reputation. **Normative beliefs** are beliefs about the extent to which other people who are important to them think they should or should not perform specific behaviours. We further distinguished between beliefs about what the employer, the supervisor, and patients expect clinical teachers to do. **Self-efficacy** beliefs are beliefs about how likely the individual perceives himself/herself to be effective in a specific task, i.e., clinical teachers’ perceived competence in providing clinical supervision. We applied the theory’s principle of the interplay between behavioural, normative, and self-efficacy beliefs in influencing the intention of clinical teachers to commit to teaching in the curriculum (due to the prospective nature of the study), accounting for a single constraint represented by language. In Southern Switzerland, Italian represents the main language spoken but English is the language of teaching at USI. We hypothesized that clinical teachers’ intention to commit themselves to teaching in the new medical curriculum will be significantly driven by these types of beliefs.

## Methods

### Study population and design

We targeted a purposive sample of physicians expected to provide clinical teaching at USI medical school. We selected all physicians working at the cantonal public hospital network (EOC), which represents the main hospital network where students receive clinical teaching. The network includes four main public hospitals and rehabilitative clinics. The administration of the EOC provided the list of all employed physicians and their e-mail addresses. No incentive or payment was offered for participation. We designed a cross-sectional quantitative study using an online questionnaire. The questionnaire, which was administered in Italian, included items developed by the authors and items adapted from a previously validated questionnaire. The questionnaire was pilot-tested for content and face validity by 15 experts with a diverse background. The piloting process proved invaluable in refining the questionnaire, as it facilitated the identification of ambiguities, redundancies, and areas for potential improvement. Based on the feedback received from the diverse panel of experts, adjustments were made to various aspects of the questionnaire, including refining the wording of certain questions, revising answer options for clarity, and incorporating additional questions to enhance our understanding of the sample.

### Questionnaire development and data collection

We developed the items of the questionnaire based on the IMBP framework, taking into account specific features and needs at a local level. Subsequently, we integrated items selected from a questionnaire focused on clinical teachers’ identity, validated in Canada [20]. Items were selected according to their relevance to the three types of beliefs described in the IMBP. One author translated all items to Italian and another author back-translated them to English to establish language equivalence. A five-point Likert scale measuring agreement (from “Strongly disagree” to “Strongly agree”) was used for all items except those measuring participants’ expectations, which asked participants whether they thought that a number of dimensions (such as their reputation) will get worse, remain stable, or improve as a result of their involvement in clinical teaching. The questionnaire was then pilot-tested for content and face validity by 15 experts including two medical educators, five experts in questionnaire development, and eight physicians with a clinical teaching role.

After minor stylistic corrections, the questionnaire was implemented using REDCap (Research Electronic Data Capture) tools hosted at USI [21, 22]. REDCap is a secure, web-based software platform designed to support data capture for research studies. To determine the extent to which the items were related to each other, we employed a model of internal consistency based on the average inter-item correlation. Here we present the value for Cronbach’s Alpha for the six subscales. The final questionnaire (supplementary file, S1) included two sections. The first section included twenty questions on participants’ sociodemographic characteristics. The second section included forty items (six subscales): three items measuring intention (α = .929), eight items measuring expectations (α = .753), ten items measuring role identification (α = .579), three items measuring the perceived importance of clinical teaching (α = .948), seven items measuring normative beliefs (α = .674), eight items measuring self-efficacy beliefs (α = .780), and one items measuring participant’s perceived linguistic barrier.

The link to the questionnaire was sent by email, together with an explanation of the nature and scope of the study. The questionnaire was accessible for 3 weeks between April 2 and 24, 2021. Written informed consent was obtained by participants before accessing the survey. One reminder was sent to all participants on April 12, 2021 to increase response rate.

### Data analysis

We used SPSS© v.24 (IBM SPSS statistics, New York, USA) for all statistical analysis. We excluded participants who did not provide written informed consent and participants who answered less than 10 questions. We conducted one-way ANOVA to compare intention, behavioural, normative, and self-efficacy beliefs, and hierarchical regression analyses to test the association between sociodemographic characteristics, beliefs and intention to commit to teaching.

## Results

### Participants characteristics

**Fig. 2:**
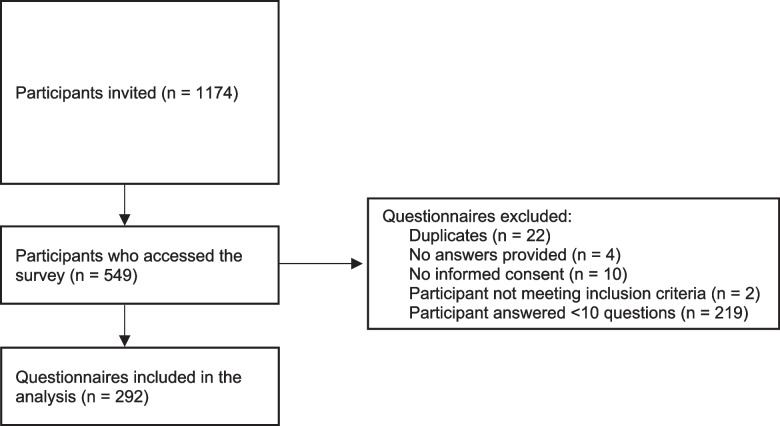
Participant flow diagram

We invited 1174 potential participants to take part in the study. Of these, 549 accessed the online survey (response rate = 46.7%) and 292 questionnaires (24.8%) were included for analysis (Fig. 2). 36% (*n* = 103) of participants were female and the mean age was 43.6 years (SD ± 9.7). One fourth (25.8%, *n* = 75) of participants obtained their medical degree up to 10 years before, 39.5% (*n* = 115) between 11 and 20 years before, and 34.7% (*n* = 101) more than 20 years before. Notably, most participants graduated from an Italian (55.8%, *n* = 163) or a Swiss university (36.3%, *n* = 106). Most participants (81.5%, *n* = 238) held a specialist degree. About 20% of participants (*n* = 54) held an academic title (e.g., “Professor”) or a research degree (e.g., “PhD”). Most participants were either resident (19.5%, *n* = 57), chief resident (26.4%, *n* = 77), attending physician (24%, *n* = 70), or head of division/department (10.6%, *n* = 31). 71.2% (*n* = 208) reported providing clinical supervision regularly and the mean number of years of experience as supervisor was 10.4 years (SD = 8.1; range = 1–36). Half (49.3%, *n* = 144) of participants declared having attended at least one faculty development course on clinical teaching. See Table 1 for participant characteristics.

**Table 1:** Participant characteristics

	Number	Percent
**Gender**		
Male	179	72.6
Female	103	36.0
Prefer not answer	4	1.4
**Age (in years)**	M = 43.6 (SD = 9.7; range = 27–70)	
**Years since medical degree**		
1–10	75	25.8
11–20	115	39.5
21–30	64	22.0
31–40	35	12.0
41–50	2	.7
**Country where medical degree was obtained**		
Switzerland	106	36.3
Italy	163	55.8
Other European country	10	3.4
Other non-European country	8	2.7
**Specialist degree**		
Yes	238	81.5
No	54	18.5
**Other certificates**		
Yes	108	37.0
No	177	60.6
**Academic title**		
None	237	81.2
PD^a^	23	7.9
PhD	12	4.1
Professor	19	6.5
**Current role**		
Scientific consultant	1	.3
Resident	57	19.5
Chief resident	77	26.4
Senior physician	110	37.8
Head of Division/Department/Institute	34	16.1
**Employer**		
EOC	282	96.6
Private Practice (self-employed)	3	1.0
**Offers regular clinical supervision**		
Yes	208	71.2
No	84	28.8
**Experience of supervision (years)**	M = 10.4 (SD = 8.1; range = 1–36)	
**Medical teaching courses attended**		
0	148	50.7
1	53	18.2
2	33	11.3
3+	58	19.9

^a^PD stands for Privatdozent/in and is an academic title conferred at some European universities, especially in German-speaking countries. This title merely denotes permission to teach and examine independently at the conferring faculty without a professorial appointment

### Intention, beliefs, and perceived barrier

#### Intention and linguistic barrier

Most participants (74.1%) reported being happy to commit themselves with teaching, that they see their role as the supervisor (60.3%), and that they intend to commit themselves to the teaching for the Master of Medicine (69.8%). For the majority of participants (65.8%), communicating with students who do not speak Italian is not an issue (Table 2). Participants reporting lower levels of commitment (who reported a mean score of ≤3 for the three questions) did not differ from participants reporting higher levels of commitment in terms of age, t(276) = − 1.238, *p* = .217, years passed since obtaining the medical degree, t(279) = − 1.349, *p* = .179, or country where medical degree was obtained, χ2 (1, *N* = 282) = .089, *p* = .766. Groups differed according to gender, with men being more likely to report higher intention, χ2 (2, *N* = 276) = 7.11, *p* = .029. Participants with an academic title or research degree, with at least one clinical teaching course attended, longer track record of supervision, and offering regular supervision were also more likely to report higher intention, χ2 (1, *N* = 281) = 14.341, p = <.001, χ2 (1, *N* = 282) = 24.158, p = <.001, t(280) = − 4.080, p = <.001, and χ2 (1, N = 282) = 33.392, p = <.001, respectively.

**Table 2:** Intention to commit oneself to the clinical teaching and perceived linguistic barrier

	N (%)	N (%)	N (%)	N (%)	N (%)
	Strongly disagree	Disagree	Neither agree nor disagree	Agree	Strongly agree
I would be happy to commit myself to teaching within the Master of Medicine	4 (1.4)	14 (4.8)	55 (18.8)	110 (37.7)	99 (33.9)
I easily identify myself with a teaching role within the Master of Medicine	4 (1.4)	25 (8.6)	83 (28.4)	102 (34.9)	68 (23.3)
I intend to commit myself within the Master of Medicine	5 (1.7)	18 (6.2)	62 (21.2)	114 (39)	83 (28.4)
Communicating with tudents who do not speak Italian is an issue for me	112 (38.4)	80 (27.4)	50 (17.1)	31 (10.6)	19 (6.5)

#### Behavioural beliefs

Concerning expectations, most participants did not expect that being a clinical teacher at the medical school will carry an improvement in the relationships with other colleagues nor in the opinion patients will have about them (Table 3). Only half of participants considered that their reputation as a physician (52.7%) and their academic career (54.5%) will improve. Notably, a majority of participants (53%) reported that the new charge of teaching will have a negative impact on the organization of their workday. A majority of participants believed that the quality of the care they offer to patients (67%) and their interprofessional relationship (81.4%) will not be modified from their involvement as clinical teachers.

**Table 3:** Expectations about the impact of one’s involvement as a clinical teacher

	N (%)	N (%)	N (%)
	… will get worse	… will not change	… will improve
My reputation as a physician...	N/A	132 (47.3)	147 (52.7)
My reputation among my colleagues...	2 (0.7)	190 (68.1)	87 (31.2)
My reputation among my patients...	N/A	190 (68.1)	89 (31.9)
The way I take care of my patients...	7 (2.5)	187 (67)	85 (30.5)
The organization of my workday...	148 (53.0)	106 (38.0)	25 (9.0)
The ease of access to databases...	2 (0.7)	179 (64.4)	97 (34.9)
My relationship with the rest of the team...	5 (1.8)	227 (81.4)	47 (16.8)
My academic career...	1 (.4)	126 (45.2)	152 (54.5)

Regarding role identification (Table 4), most participants found that their role as a teacher give them a lot of satisfaction (71.2%). They envisioned their role as the one of transmitting their experience (63.8%) and agreed about the importance of feedback as a learning tool (85.8%). For most participants, the progress of residents/students depends first and foremost on their motivation (77%), residents’/students’ wellbeing is the priority(59%), and it is important to explain the how and why when demonstrating how to perform clinical tasks (97.6%). Most participants did not see their role as a companion (52.1%) nor as that of an older brother/sister (51.8%), and did not considered their resident as a student (56.5%).

**Table 4:** Self-identification in the role of a clinical teacher

	N (%)	N (%)	N (%)	N (%)	N (%)
	Strongly disagree	Disagree	Neither agree nor disagree	Agree	Strongly agree
It’s important to check whether a student/resident meets the expected level of performance at each stage of his/her training	1 (.4)	1 (.4)	12 (4.2)	168 (59.4)	101 (35.7)
My role as a teacher gives me a lot of satisfaction	2 (.7)	11 (3.9)	68 (24.2)	136 (48.4)	64 (22.8)
The best learning comes from receiving relevant and useful feedback from the teacher	N/A	4 (1.4)	36 (12.7)	175 (61.8)	68 (24)
The progress of residents/students depends first and foremost on their motivation (R)	N/A	6 (2.1)	59 (20.8)	174 (61.5)	44 (15.5)
I want my residents/students to feel good rather than to stress them	2 (.7)	24 (8.5)	90 (31.8)	126 (44.5)	41 (14.5)
The most important thing we need to pass on to residents/students is our experience	3 (1.1)	18 (6.4)	81 (28.7)	139 (49.3)	41 (14.5)
When you show residents/students how to do things, it is important to explain how and why you are doing them	1 (.4)	1 (.4)	5 (1.8)	140 (49.5)	136 (48.1)
With respect to my residents/students, I see my role as a companion	33 (11.6)	115 (40.5)	93 (32.7)	36 (12.7)	7 (2.5)
With respect to my residents/students, I see my role as that of an older brother/sister	40 (14.1)	107 (37.7)	74 (26.1)	57 (20.1)	6 (2.1)
I consider my resident as a student.	40 (14.2)	119 (42.3)	93 (33.1)	27 (9.6)	2 (.7)

In terms of perceived importance (Table 5), most participants agreed or strongly agreed that doing clinical supervision is significant to them (82.3%), that clinical supervision is meaningful to them (81.7%), and that they have clinical supervision at heart (81.3%).

**Table 5:** Perceived importance

	N (%)	N (%)	N (%)	N (%)	N (%)
	Strongly disagree	Disagree	Neither agree nor disagree	Agree	Strongly agree
Doing clinical supervision is significant to me	1 (.3)	5 (1.7)	45 (15.6)	140 (48.4)	98 (33.9)
Clinical supervision is meaningful to me	1 (.3)	7 (2.4)	45 (15.6)	143 (49.5)	93 (32.2)
I have clinical supervision at heart	2 (.7)	8 (2.8)	44 (15.2)	127 (43.9)	108 (37.4)

#### Normative beliefs

Only a minority of respondents reported that patients, the employer, or relevant political institutions believe that their commitment to teaching at the medical school will have an impact on the quality of care (Table 6). They reported, indeed, that caring for patients is what is most expected of them (76.2%).

**Table 6:** Normative beliefs

	N (%)	N (%)	N (%)	N (%)	N (%)
	Strongly disagree	Disagree	Neither agree nor disagree	Agree	Strongly agree
My direct supervisor believes that my commitment to teaching in the Master of Medicine program can make a difference on the quality of care	16 (5.5)	17 (5.9)	137 (47.2)	78 (26.9)	42 (14.5)
My patients believe that my commitment to teaching in the Master of Medicine program can make a difference on the quality of care	20 (6.9)	30 (10.3)	167 (57.6)	57 (19.7)	16 (5.5)
My institution believes that my commitment to teaching in the Master of Medicine program can make a difference on the quality of care	15 (5.2)	18 (6.2)	112 (38.8)	106 (36.7)	38 (13.1)
The Cantonal Department of Health and Social Welfare believes that my commitment to teaching in the Master of Medicine program can make a difference on the quality of care	10 (3.5)	17 (5.9)	168 (58.5)	73 (25.4)	19 (6.6)
We need to hold regular case discussion sessions with our residents/students	6 (2.1)	12 (4.2)	33 (11.6)	155 (54.4)	79 (27.7)
As far as I know, none of the supervisors I know observe the work of their residents/students	96 (33.3)	113 (39.2)	73 (25.3)	6 (2.1)	N/A
Caring for patients is what is most expected of me	3 (1.0)	20 (6.9)	46 (15.9)	138 (47.6)	83 (28.6)

#### Self-efficacy beliefs

Participants showed high self-efficacy beliefs (Table 7). A large majority of them were confident about their skills as a clinical supervisor (78.7%), and 61.3% even considered themselves as being proficient in these skills. They consider they can help both good and less good students to improve (82.4%), and that their teaching could have an impact on the progression of their learners (80.4%).

**Table 7:** Self-efficacy

	N (%)	N (%)	N (%)	N (%)	N (%)
	Strongly disagree	Disagree	Neither agree nor disagree	Agree	Strongly agree
I am confident about my skills as a clinical supervisor	N/A	7 (2.4)	55 (18.9)	173 (59.5)	56 (19.2)
I am completely proficient in the skills that are necessary for clinical supervision	N/A	27 (9.2)	86 (29.5)	141 (48.3)	38 (13.0)
I am confident in my ability to perform clinical supervision	N/A	19 (6.6)	67 (23.3)	154 (53.5)	48 (16.7)
I play it by ear when I try to help my residents/students	61 (21.0)	108 (37.1)	75 (25.8)	41 (14.1)	6 (2.1)
I can help a good resident/student become even better, but there is nothing I can do for bad ones	96 (33.1)	143 (49.3)	37 (12.8)	11 (3.8)	3 (1.0)
Once in specialized training, most residents do not need us to intervene	135 (46.4)	137 (47.1)	13 (4.5)	6 (2.1)	N/A
I don’t see what I can bring to my residents/students	183 (63.1)	90 (31.0)	13 (4.5)	3 (1.0)	1 (.3)
I don’t know whether my interventions have any impact on the progression of my residents/students	113 (38.8)	121 (41.6)	44 (15.1)	12 (4.1)	1 (.3)

### Regression model

We estimated a regression model of socio-demographic characteristics, behavioural, normative, and self-efficacy beliefs, and the intention to commit themselves to the teaching for the Master of Medicine (Table 8). We found that following factors were positively and significantly associated with intention to commit to the clinical teaching: having an academic title or research degree, the number of clinical teaching training sessions attended, self-efficacy, perceived importance, and expectations. Gender, age and years of regular supervision were not significantly correlated with intention. The model explained 53% of the total variance.

**Table 8:** Regression model for the intention to commit to teaching

Variable	Standardized beta	*t*	*p*
Age	−.005	−.853	.394
Gender	.018	.207	.836
Years of regular supervision	.009	1.289	.199
**Having an academic title or research degree**	**.277**	**2.417**	**.016**
**Number of teaching courses attended**	**.087**	**2.444**	**.015**
Perceived linguistic barrier	−.001	−.032	.975
Social norms	.003	.240	.811
**Self-efficacy**	**.301**	**3.057**	**.002**
**Perceived importance**	**.463**	**5.932**	**<.001**
Role identification	.092	.730	.466
**Expectations**	**.707**	**4.674**	**<.001**
R2	.53		

## Discussion

High quality training of the future healthcare workforce is fundamental to ensure the delivery of safe and effective patient care [23]. On the establishment of a new master school of medicine, we explored beliefs about teaching and intention to commit to teaching among a purposive sample of prospective clinical teachers using the Integrative Model of Behaviour Prediction (IMBP) of Fishbein [19] as framework. Since beliefs are important factors influencing behaviours, the results of our study allow to gain deeper insight to predict the intention to teach of our future teachers [19].

We found that most participants displayed high intention to commit to clinical teaching and could easily identify themselves in this role. This intention was positively associated to being on an academic track and having attended one or more faculty development programs for clinical teaching. As postulated by the IMBP, the intention to commit with teaching appears also to arise from positive self-efficacy beliefs about proficiency in clinical teaching and from some behavioural beliefs (expecting benefits from clinical teaching and attributing a high importance to clinical teaching). A large majority of participants do not expect their involvement as teacher to negatively affect the quality of care provided to patients nor their interprofessional relationships, which may even improve for some of them.

On the other hand, we found that participants held widespread normative beliefs and negatives expectations that could be potential restraining factors to the intention to teach in the new medical school curriculum. In this regard, the majority of participants did not anticipate any significant changes in their reputation nor tangible benefits resulting from their role as teachers. Furthermore, half of the participants did not expect any enhancement in their academic career, but rather expressed concerns about the potential additional workload and its impact on their work organization. These findings indicate a prevailing perception that teaching is not adequately rewarded, neither within their healthcare institutions nor by the university. This observation raises a critical point of concern, which resonates with similar patterns observed by other authors [24].

While the initial phase of enthusiasm and novelty surrounding the establishment of a new school may generate motivation among educators, in the long term the lack of recognition and rewarding for teaching efforts can potentially hinder the intention to engage in teaching activities. This situation is particularly troubling in a curriculum where teaching in the clinical settings constitutes a substantial portion of the instructional activities, as was the focus of our study.

We found normative beliefs, revealing a certain disillusionment, originating from a disparity between the self-perceived value of teaching and the expectations placed on clinical teachers by various stakeholders. This disillusionment could originate from the awareness that the prevailing role attributed to physicians, by the society and health care institutions, essentially revolves around patient care, with teaching often confined to a subordinate level of importance [5, 25].

Echoing another concern noted by participants, it is essential to ensure that teachers are provided with protected time for their teaching responsibilities [26–28]. Back in 2006, the Board of Medical Education of the British Medical Association identified the lack of recognition and rewards, the lack of support and the time pressure as the main challenges facing medical teachers [29]. Our prospective teachers seem to anticipate the “triple invisibility” of physicians as educators described by Sabel et al., which combine the lack of recognition, with the lack of clear career pathway and the lack of identity [30]. As long as the perceived value of educators lags behind that of clinical service provision and clinical research, medical education will continue to be perceived as the Cinderella of all medical disciplines [30, 31]. To ensure high-quality medical training and patient care, it is therefore crucial recognize and encourage teachers’ commitment at an appropriate level [23]. Allsop et al. recently proposed a model for a systematic approach to re-frame a culture which values medical education in terms of adequate funding and full, supportive stakeholders involvement [23].

Our results finally confirm the central role and the potential benefits of specific targeted faculty development programs to support and enhance teachers’ commitment to teaching, which, in turn could lead to improved educational outcomes for students. Previous studies indeed demonstrated a positive association between attendance to faculty development programs and teaching effectiveness [32, 33].

The present study provides partial support for Fishbein’s Integrative Model of Behavior Prediction in the context of clinical teaching. The regression analysis showed that only three model’s constructs (self-efficacy, perceived importance and expectations) significantly predict intention to commit to teaching This finding is important as previous research has shown how confident teachers are more likely to employ effective teaching styles, leading to enhanced educational outcomes [34].

The identification of three specific constructs—self-efficacy, perceived importance, and expectations—as significant predictors of intention to commit to teaching in the context of clinical teaching within Fishbein’s Integrative Model of Behavior Prediction raises some considerations. Notably, the selective influence of these constructs suggests a nuanced interplay between psychological factors that motivate and shape clinical teachers’ commitment to teaching. The theoretical underpinning of the Integrative Model implies that various constructs collectively contribute to behavioural intention. However, the specific emphasis on self-efficacy, perceived importance, and expectations in this study underscores their heightened relevance within the context of clinical teaching.

The emergence of these three constructs as significant predictors could be attributed to their direct impact on the motivational and cognitive processes governing teachers’ commitment. Self-efficacy reflects an individual’s belief in their ability to perform a specific task successfully. In the context of clinical teaching, a teacher’s confidence in their instructional abilities may crucially influence their intention to commit to the role. Perceived importance underscores the subjective significance an individual attaches to a particular behaviour, indicating that educators who perceive teaching as highly important are more likely to express a commitment to it. Expectations, as a construct, encapsulate the anticipated outcomes and consequences of one’s actions, providing insight into the extrinsic motivational drivers behind the commitment to teaching.

The alignment of these three constructs with the chosen theoretical model suggests that, within the framework of Fishbein’s Integrative Model, these factors hold particular salience in predicting intentions related to clinical teaching. It implies that, while other factors may play a role, the unique dynamics of the clinical teaching context elevate the relevance of self-efficacy, perceived importance, and expectations as primary drivers of teachers’ commitment.

To further support clinical teachers’ intentions, it becomes essential to explore the sources of their self-efficacy, perceived importance, and expectations. Existing research provides insights into how mentoring programs, professional development initiatives, and positive experiences in teaching practice contribute to the enhancement of self-efficacy among medical educators [35–37]. Additionally, understanding the contextual factors that shape perceived importance and expectations—such as institutional support, recognition, and the broader educational environment—can inform strategies to cultivate and sustain educators’ commitment to teaching in clinical settings. By addressing these factors comprehensively, educational institutions can foster an environment conducive to the positive intentions and sustained commitment of clinical teachers.

The notion of self-efficacy deserves specific emphasis. The concept of perceived self-efficacy, as distinguished from actual efficacy, has been a subject of interest in psychology and education literature. Perceived self-efficacy refers to an individual’s beliefs and confidence in their own ability to perform a specific task or achieve a particular outcome. On the other hand, actual efficacy involves an objective assessment of an individual’s performance in the given task. The disjunction between perceived and actual efficacy is well-documented in the psychological literature [38, 39]. While perceived self-efficacy can be a strong motivator and predictor of engagement, it does not always align with actual competence. Several studies in the field of psychology have explored this phenomenon across various domains. In the context of teaching, research has shown instances where some individuals may exude confidence in their ability to teach, yet their actual teaching effectiveness may not meet the expectations associated with their perceived self-efficacy [40].

This discrepancy between perceived and actual efficacy underscores the importance of a comprehensive assessment that includes objective measures of performance. It also highlights the need for ongoing professional development and reflective practices to ensure that educators continually enhance their actual teaching skills, aligning them more closely with their perceived self-efficacy. he discrepancy between self-efficacy and actual efficacy in human behavior arises from factors such as overestimation of abilities, limited feedback, task complexity, cognitive dissonance, emotional influences, and social comparisons. Individuals may overestimate their competence due to cognitive biases or lack of accurate self-assessment. The absence of external evaluation and feedback can contribute to misalignments. Task complexity impacts self-efficacy judgments, and cognitive dissonance leads to the preservation of positive self-images. Emotional states and motivation influence self-efficacy, and social comparisons can distort perceptions. Additionally, contextual factors, like the teaching environment, affect self-efficacy beliefs independently of actual teaching effectiveness. Understanding and addressing this disjunction can contribute to the improvement of teaching quality and the effectiveness of educational practices.

Interestingly, role identification and normative beliefs were not found to be significantly associated with the intention to commit to clinical teaching. Role identity was measured using items reflecting a proactive view of supervisors’ roles, incorporating merged identities as both clinicians and teachers. This suggests that other factors beyond role identity and normative beliefs may play a more influential role in shaping teaching commitment among clinical educators [20].

Our study has a number of limitations. First, given the low response rate, we cannot exclude that results have been susceptible to self-selection bias. The length of the survey, the web-only delivery of the survey and the lack of financial incentives could have play a role [41]. Nevertheless, the low response rate among physician is a well-known phenomenon and our study do not differ from other surveys sent to physicians [42]. Second, given the population sample restricted to a specific geographical region, our results could not be generalizable to other contexts. Nevertheless our experience within other well established medical school as well as the literature [23] suggest that our results may be also relevant to other contexts.

### Implications

This study provides useful insights into the beliefs of prospective teachers at a new medical school, allowing them to be specifically addressed. Some aspects could be easily addressed with specific faculty development courses, such targeted techniques for carrying out time-efficient clinical teaching, or the correction of some normative beliefs, e.g. illustrating existing possibilities for the valorisation of teaching activities. Other aspects, such as rewarding teaching activities, promoting academic careers in medical education, and ensuring protected time for teaching, will need to be discussed at a higher level with policy, university governance, and health care institutions. In addition, sessional commitments to teaching (teaching responsibilities and workload of sessional or adjunct faculty members, which may differ from those of full-time, tenured, or tenure-track professors) should be clarified [43], and clinical teachers should be offered personalized coaching [44].

### Supplementary Information

**Additional file 1.** Supplementary Material 1

## Data Availability

The datasets used and/or analysed during the current study are available from the corresponding author on reasonable request.
