# Supplementary material for: Exploring clinical teachers’ beliefs about teaching in a newly established medical school in Southern Switzerland
Source: BMC Med Educ. 2024 Mar 22;24:330. doi: 10.1186/s12909-024-05299-0 (PMC10960412; doi:10.1186/s12909-024-05299-0)
Supplement: Supplementary file 1 — Additional file 1. Supplementary Material 1 [file 12909_2024_5299_MOESM1_ESM.docx]

**Supplement 1.** Questionnaire items

|  | Item | Scale and anchors |
| --- | --- | --- |
| **Intention** |  |  |
| Q1 | I would be happy to commit to the teaching within the Medical Master school | Five-point Likert scale ranging from “Strongly disagree” to “Strongly agree” |
| Q2 | I intend to commit myself within the Medical Master school |  |
| Q3 | It is easy for me to identify with a teaching role within the Medical Master school at USI |  |
|  |  |  |
| **Behavioural beliefs** | |  |
| Expectations | | |
| Q4 | My reputation as a physician... | Three options: “Will improve”, “Will worsen”, “Will not change” |
| Q5 | My colleagues' opinion on me... |  |
| Q6 | My patients' opinion on me... |  |
| Q7 | My taking care of my patients... |  |
| Q8 | The organization of my workday... |  |
| Q9 | The ease of access to databases... |  |
| Q10 | My relationship with the rest of the team... |  |
| Q11 | My academic career... |  |
|  |  |  |
| Role identification | |  |
| Q12 | It is important to make sure that an resident/student meets the expected level of performance at each stage of his or her training* | Five-point Likert scale ranging from “Strongly disagree” to “Strongly agree” |
| Q13 | My teaching role gives me a lot of satisfaction* |  |
| Q14 | The best learning comes from receiving relevant and useful feedback* |  |
| Q15 | The progress of residents/students depends first and foremost on their motivation* (R) |  |
| Q16 | I want my residents/students to feel good rather than to stress them* |  |
| Q17 | The most important thing we need to pass on to residents/students is our experience* |  |
| Q18 | When you show residents/students how to do things, it is important to explain how and why you are doing them* |  |
| Q19 | With respect to my residents/students, I see my role as a companion* |  |
| Q20 | With respect to my residents/students, I see my role as that of an older brother/sister* |  |
| Q21 | I consider my resident as a student* |  |
|  |  |  |
| Perceived importance of clinical teaching | | |
| Q22 | Doing clinical supervision is significant to me | Five-point Likert scale ranging from “Strongly disagree” to “Strongly agree” |
| Q23 | Clinical supervision is meaningful to me |  |
| Q24 | I have clinical supervision at heart |  |
|  |  |  |
| **Normative beliefs** | |  |
| Q25 | My direct supervisor believes that my commitment to teaching in the Master of Medicine program can make a difference on the quality of care | Five-point Likert scale ranging from “Strongly disagree” to “Strongly agree” |
| Q26 | My patients believe that my commitment to teaching in the Master of Medicine program can make a difference on the quality of care |  |
| Q27 | My institution (e.g., EOC) believes that my commitment to teaching in the Master of Medicine program can make a difference on the quality of care |  |
| Q28 | The Cantonal Department of Health and Social Welfare (DSS) believes that my commitment to teaching in the Master of Medicine program can make a difference on the quality of care |  |
| Q29 | We have to organize regular sessions of case discussions with our residents* |  |
| Q30 | To my knowledge, none of the supervisors I know observe their residents* (R) |  |
| Q31 | Treating patients is what is most expected of me* (R) |  |
|  |  |  |
| **Self-efficacy beliefs** | | |
| Q32 | I am confident about my skills as a clinical supervisor | Five-point Likert scale ranging from “Strongly disagree” to “Strongly agree” |
| Q33 | I completely master the skills that are necessary for clinical supervision |  |
| Q34 | I am confident in my ability to perform clinical supervision |  |
| Q35 | I play it by ear when I try to help my residents/students* (R) |  |
| Q36 | I can help a good resident/student become even better, but there is nothing I can do for bad ones* (R) |  |
| Q37 | Once in specialized training, most residents do not need us to intervene* (R) |  |
| Q38 | I don't really see what I can bring to my residents/students* (R) |  |
| Q39 | I don’t know whether my interventions have any impact on my residents’ progression* (R) |  |
|  |  |  |
| **Perceived linguistic barrier** | |  |
| Q40 | Communicating with interns/students who do not speak Italian is an issue for me | Five-point Likert scale ranging from “Strongly disagree” to “Strongly agree” |

* Items adapted from Dory et al., 2015

(R) = Reversed item
